# Unraveling the whole genome DNA methylation profile of zebrafish kidney marrow by Oxford Nanopore sequencing

**DOI:** 10.1038/s41597-023-02431-5

**Published:** 2023-08-10

**Authors:** Xudong Liu, Ying Ni, Dandan Wang, Silin Ye, Mengsu Yang, Xuan Sun, Anskar Yu Hung Leung, Runsheng Li

**Affiliations:** 1 Department of Infectious Diseases and Public Health, Jockey Club College of Veterinary Medicine and Life Sciences, City University of Hong Kong, Hong Kong, China; 2 Department of Precision Diagnostic and Therapeutic Technology, City University of Hong Kong Shenzhen Futian Research Institute, Shenzhen, China; 3 Department of Biomedical Sciences and Tung Biomedical Sciences Centre, City University of Hong Kong, Hong Kong, China; 4 Division of Haematology, Department of Medicine, LKS Faculty of Medicine, The University of Hong Kong, Pokfulam, Hong Kong SAR China; 5 Key Laboratory of Biochip Technology, Biotech and Health Centre, Shenzhen Research Institute of City University of Hong Kong, Shenzhen, China; 6 ZeBlast Technology Limited, Hong Kong Science Park, Hong Kong, China; 7 Southern Marine Science and Engineering Guangdong Laboratory (Guangzhou), Guangzhou, China

**Keywords:** Data mining, Genetic databases

## Abstract

Zebrafish is a widely used model organism for investigating human diseases, including hematopoietic disorders. However, a comprehensive methylation baseline for zebrafish primary hematopoietic organ, the kidney marrow (KM), is still lacking. We employed Oxford Nanopore Technologies (ONT) sequencing to profile DNA methylation in zebrafish KM by generating four KM datasets, with two groups based on the presence or absence of red blood cells. Our findings revealed that blood contamination in the KM samples reduced read quality and altered methylation patterns. Compared with whole-genome bisulfite sequencing (WGBS), the ONT-based methylation profiling can cover more CpG sites (92.4% vs 70%–80%), and exhibit less GC bias with more even genomic coverage. And the ONT methylation calling results showed a high correlation with WGBS results when using shared sites. This study establishes a comprehensive methylation profile for zebrafish KM, paving the way for further investigations into epigenetic regulation and the development of targeted therapies for hematopoietic disorders.

## Background & Summary

Zebrafish (*Danio rerio*) is a widely used model organism in biomedical research, particularly for studying human diseases such as cancer^1^, heart disease^2^, and blood-related disorders^3^. This is due to their genetic and physiological similarities to human^4,5^. Comparative genomic analysis has shown that the zebrafish genome shares a high degree of synteny and sequence homology with the human genome, with approximately 70% of human genes having at least one clear zebrafish orthologue^6,7^.

The hematopoietic system is responsible for producing and maintaining blood cells in the animal body. In adult zebrafish, the kidney marrow (KM) has been identified as the primary hematopoietic organ where hematopoietic stems differentiate into various types of blood cells including erythrocytes, leukocytes, and thrombocytes^8,9^. The zebrafish kidney marrow is considered to be analogous to the mammalian bone marrow^8^. Therefore, zebrafish KM has become an important model for studying hematopoiesis and blood-related discorders^10,11^. Unlike mammalian red blood cells, zebrafish red blood cells are nucleated and DNA-rich. Consequently, when extracting KM DNA in zebrafish, red blood cells can represent a significant fraction of the samples, potentially impacting data interpretation for downstream analyses, including quality control and DNA methylation profiling.

Epigenetic modifications, such as DNA methylation, are heritable changes capable of modulating gene expression in hematopoietic disorders. Aberrant DNA methylation patterns have been observed in various hematologic malignancies, including leukemia^12^. These alterations can result in dysregulated gene expression patterns, which contribute to the initiation and progression of these disorders. For example, hypermethylation of tumor suppressor genes can lead to their silencing, promoting cancer development^13^.

Investigating the role of DNA methylation in hematopoietic disorders using the zebrafish KM model is crucial for developing novel diagnostic and therapeutic strategies for human blood-related diseases. However, the absence of a comprehensive methylation baseline in wild-type zebrafish KM remains a challenge for achieving this goal.

The gold standard for detecting the 5-methylcytosine (5mC) methylation with high throughput sequencing still relies on the bisulfite conversion. Whole-genome bisulfite sequencing (WGBS) can be used to detect the 5mC methylation on a genome-wide scale. However, the bias arises from chemical treatments, and subsequently PCR amplification hindered the detection of 5mC levels in some specific regions of the genome. A recently published WGBS data for different zebrafish tissues^14^ is based on GRCz10 assembly, while the most recently updated genome assembly is GRCz11. A liftover of these datasets to new assembly are needed to make the dataset more accessible to the zebrafish research community.

Using nanopore sequencing technology to detect DNA 5mC methylation offers several significant advantages, including long-read sequencing, direct detection of base modifications, and single-molecule resolution^15^. As a long-read sequencing platform, the read GC bias could be less than NGS, which is a result of amplification. Nanopore sequencing can directly detect 5mC modifications by inferring the raw current signal. This eliminates the need for additional chemical treatments, reducing the risk of DNA damage and minimizing potential biases introduced during the conversion process. Moreover, nanopore sequencing provides single-molecule resolution, enabling the detection of 5mC methylation patterns at individual DNA molecules. This allows for the identification of rare and heterogeneous methylation events, which may be crucial for understanding the functional consequences of DNA methylation in different biological contexts.

In this study, we employed the Oxford Nanopore Technologies (ONT) platform to profile DNA methylation in the zebrafish KM with or without red blood cells and compared the genome-wide 5mC profiling capacity using ONT reads with the WGBS method. This dataset establishes a foundation for further exploration of the epigenetic regulation of hematopoietic disorders and the development of targeted therapeutic interventions in zebrafish.

## Methods

### Sample collection and DNA extraction

Wild-type Tubingen (TU) zebrafish lines were obtained from the Zebrafish International Resource Center (ZIRC, USA). The study received approval from the Committee on the Use of Laboratory and Research Animals (CULATR), approval number CULATR 5649-21, at the University of Hong Kong (HKU). Kidney marrow from 8-month-old WT zebrafish was collected, dissociated in 0.9X PBS via pipetting, and filtered through a 40 µm nylon cell strainer (Corning, NY, USA). Some samples underwent treatment with eBioscience™ 1x RBC Lysis Buffer (Invitrogen, MA, USA) to lyse red blood cells, and these samples were designated as the KM group. Samples without red blood cell lysis were categorized as the KMB group. The resulting cells were centrifuged at 500 g for 5 minutes, resuspended, filtered, and washed twice with 0.9X PBS for DNA extraction. Genomic DNA was extracted using QIAamp DNA Kits (Qiagen, Germany).

### Library preparation and nanopore sequencing

Approximately 2 μg of Genomic DNA extracted from the kidney marrow cells of zebrafish was used to prepare the library with a ligation sequencing kit (LSK-110, ONT). The libraries were sequenced on MinION Mk1B after loading to R9.4.1 flow cells (FLO-MIN106D, ONT).

### Read processing

The generated raw Nanopore data (fast5 type) were basecalled by Dorado v. 0.1.1 (https://github.com/nanoporetech/dorado) with the dna_r9.4.1_e8_sup@v3.3 super-accuracy model. Adapters from nanopore reads were removed by Porechop v. 0.2.4 (https://github.com/rrwick/Porechop) and both head and tail 50 bp bases were cut off for each read using NanoFilt v.2.8.0^16^. Reads with a low length than 200 bp and an estimated read quality score below 7 were removed by NanoFilt v.2.8.0^16^.

### Read quality analysis

The reads were aligned to the zebrafish reference (GRCz11) using Minimap2 v.2.22^17^ with the argument “–secondary = on”. To summarize the read accuracy of different libraries, we counted the estimated accuracy (Eq. 2) and observed accuracy (Eq. 3) for each aligned read:

1 $$N\left(total\right)=N\left(sub\right)+N\left(mat\right)+N\left(ins\right)+N\left(del\right)$$

2 $$Estimated\;read\;accuracy=1-\left[\frac{1}{N}\ast \sum 1{0}^{-{q}_{i}/10}\right]$$

3 $$Observed\;read\;accuracy=N(mat)/N(total)$$

Here, *N* was the number of the base in each read and *q*_*i*_ was the *i*-th basecalled base quality score. *N(sub)*, *N(mat)*, *N(ins)*, and *N(del)* were the number of substitution(s), match(es), insertion(s), and deletion(s) in each read, respectively.

### Methylation profiling

All the raw Nanopore data (fast5 type) were basecalled by Bonito v.0.6.1 (https://github.com/nanoporetech/bonito) with the dna_r9.4.1_e8_sup@3.3 model and the argument of “--modified_bases 5mc” for 5mC to *bam* files with methylation information. To get the site-level modification proportion, modbam2bed v.0.6.3 (https://github.com/epi2me-labs/modbam2bed) was used to profile the bam file. To obtain the proportion of covered CpG sites in ONT methods, only the site that had at least one forward and one reverse sequencing read coverage was counted. To assess the methylation status of each chromosome region, the chromosome was divided into 100Kb fragments. The 5Kb fragments upstream of the transcription start site (TSS) were defined as the promoter region. The regional methylation proportion was then calculated for each fragment using an in-house script, which can be found at https://github.com/lrslab/Zebrafish-Multisequencing.

### Dataset subsampling and coverage estimation

To evaluate the reference coverage of the datasets, we implemented a subsampling approach using the seqkit^18^ tool with the “sample” argument, generating subsets of the dataset at ten different proportions, spanning a range from 0% to 100%. These subsets were then aligned to the reference using minimap2^17^ with the “-ax map-ont” argument, and the resulting alignments were used to calculate the coverage of the reference using the samtools^19^ tool.

### GC bias comparison

To compare the GC bias of whole-genome bisulfite sequencing (WGBS) and Oxford Nanopore Technologies (ONT) datasets, we mapped the datasets to the zebrafish genome (GRCz11) using bwa and minimap2^17^ for NGS and ONT data, respectively. We utilized samtools^19^ to profile the genome coverage at the 1k bin level using the “bedcov” argument. The resulting bins were then classified based on their GC content, which ranged from 0% to 100%. We subsequently calculated the read coverage for each GC content level and determined the average read coverage for GC content ranging from 10% to 60%. We normalized the read coverage by dividing the coverage of each GC content level within this range by the overall average read coverage.

## Data Records

The raw sequencing reads and methylation *bam* files of all samples are available from the NCBI via the BioProject accession number PRJNA930374^20^. The ONT regional level methylation profiling on zebrafish GRCz11 assembly can also be found in Gene Expression Omnibus (GEO) with accession number GSE232842^21^. The WGBS per-site, gene promoter, and 100 Kb bins level methylation profiling files on zebrafish GRCz11 assembly generated from the GRCz10 assembly using LiftOver are available at the Figshare with the DOI number (10.6084/m9.figshare.22689700^22^ and 10.6084/m9.figshare.22785191^23^).

## Technical Validation

### Read statistics

We presented four datasets of zebrafish samples collected from kidney marrow (KM). The samples were divided into two groups based on the sample handling process. The first group contained kidney marrow with blood (KMB), while the second group filtered out the blood DNA by adding a red blood cell lysis before the DNA extraction. Both groups of samples were sequenced by Oxford Nanopore Technologies (ONT) platform, with one R9.4.1 flow cell used for each sample. The raw yield for KMB1, KMB2, KM1, and KM2 was 7.2 Gb, 9.8 Gb, 20.6 Gb, and 20.5 Gb, respectively (Supplementary Table 1). To obtain clean data, several filters were applied, including adapter, short read, and low quality read removal (see methods for detail). After filtering, at least 85% of high-quality reads were retained for further analysis (Supplementary Table 1).

### Read accuracy and genome recovery rate

**Fig. 1:**
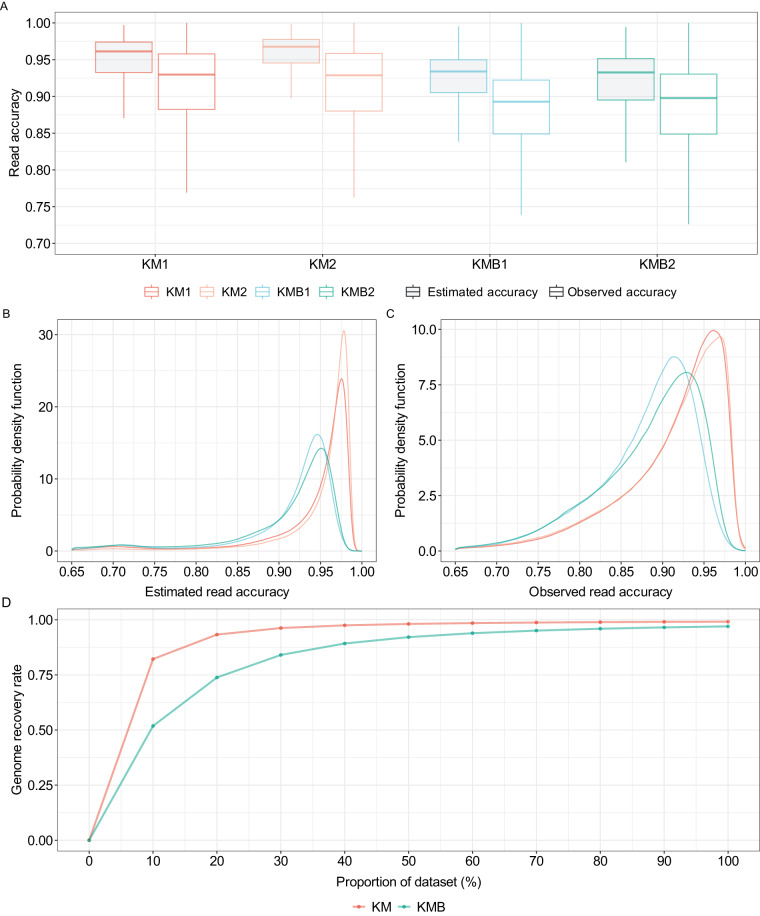
Quality assessment of nanopore reads obtained from whole-genome shotgun (WGS) sequencing using R9.4.1 flow cells. The four sample datasets, KM1, KM2, KMB1, and KMB2, are categorized into two groups: kidney marrow (KM) and kidney marrow with blood (KMB). (**A**) Comparison of estimated read accuracy (grey) and observed read accuracy (white) for the four sample datasets. (**B**) Density distribution of estimated read accuracy. (**C**) Density distribution of observed read accuracy for mapped reads. (**D**) Genome recovery rate of the two combined datasets, KM and KMB.

High read accuracy is of paramount importance for downstream analysis and obtaining accurate results. To accurately assess the quality of the four datasets, we calculated both the estimated read accuracy and observed read accuracy. The average estimated accuracy for the two datasets in the KMB group was approximately 92.5%, while the two datasets in the KM group reached 95% (Fig. 1A). The average observed read accuracies for KMB and KM samples are approximately 90% and 92.5% (Fig. 1A). The modal read accuracy provides results similar to those obtained from the average read accuracy that the read quality for KM is better than that for KMB. The modal read accuracy for KM1 and KM2 exceeded 95%, while those for KMB1 and KMB2 were below 95% (Fig. 1B,C). To exclude the potential influence of read length to sequencing quality across different groups, we conducted a comparative analysis of read length and observed read accuracy. Specially, we computed the correlation coefficients between these two variables in four samples. Our results indicated that the absolute value of Pearson correlation coefficients (R) in all four samples were less than 0.2, suggesting no significant association between read length and read accuracy (Fig. S1). To account for differences in read coverage across the samples, which may affect the quality of sequencing data, we further subsampled the yield of KM1, KM2 and KMB2 to match that of KMB1 (7.2 Gb). We then compared the estimated and observed read accuracy for each sample. Our results were consistent with those obtained using the total reads (Fig. S2). It should be noted that the zebrafish DNA sequencing data was not used for training Nanopore basecalling models, which may result in a lower observed read accuracy when compared to human sequencing data^24^.

To calculate the genome recovery rate for each group, we merged the dataset within the same group and subsampled the reads. The KMB reads covered 96.7% of the GRCz11 genome and KM reads covered 99.1% when using the full dataset (Fig. 1D). The reduced genome recovery rate is related to the low read yield in KMB samples.

Our evaluation of read accuracy for the KM and KMB groups revealed that both the estimated and observed read accuracy were consistently lower in the KMB group when compared to the KM group. These results suggest that the presence of red blood cells in the kidney marrow samples may have contributed to the lower read quality during the sequencing.

### Methylation pattern

**Fig. 2:**
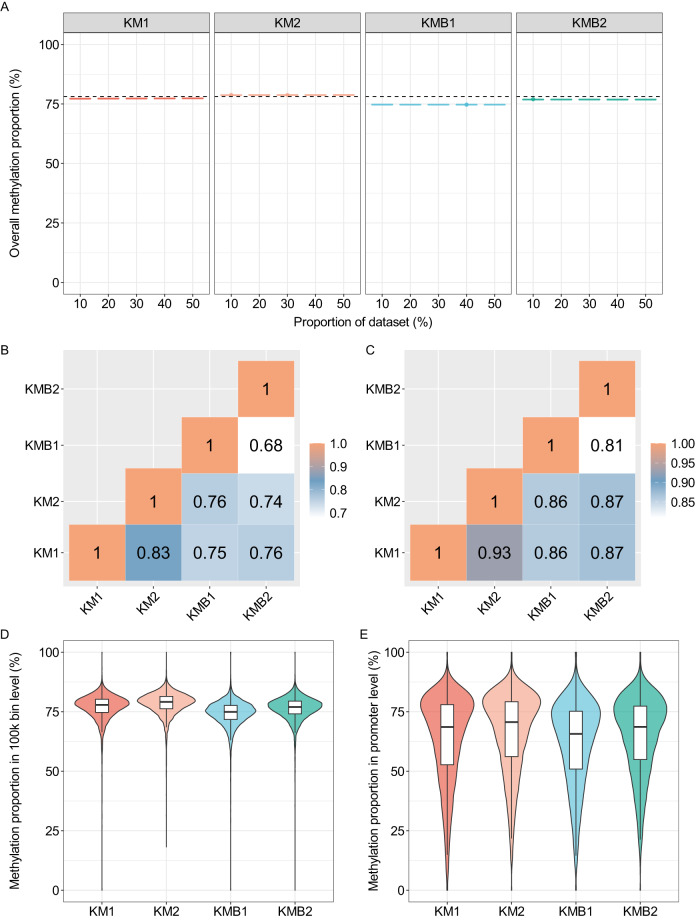
The ONT based methylation profiling from the four datasets. (**A**) Robustness of methylation calling across the whole genome assessed by subsampling total reads into different proportions. Results were obtained by subsampling reads to specific proportions 10 times. The dashed line represents the average 5mC proportion for kidney marrow (78.2%). (**B**) Pearson correlation coefficients between the four datasets in the 100 kb bin intervals. (**C**) Pearson correlation coefficients between the four datasets in the promoter regions. (**D**) Distribution of the four datasets at the 100 kb bin intervals. (**E**) Distribution of the four datasets at the promoter level.

Given the critical role of base methylation in diverse disease mechanisms, we sought to investigate whether blood contamination would have an impact on the methylation information of our kidney marrow samples. To ensure that our read depth was sufficient for accurate 5mC methylation calling, we conducted a subsampling analysis on each dataset. Specifically, we randomly subsampled each dataset to different proportions and repeated this process 10 times for each proportion. The overall methylation for each subsampled dataset was calculated and results demonstrate that each dataset exhibits stable methylation patterns across all subsampling proportions, indicating that the read depth is sufficient for accurate methylation calling (Fig. 2A). The overall 5mC methylation proportion of subsampling datasets for KMB1 and KMB2 were 74.7% and 76.8%, respectively, which were lower than that of the kidney marrow dataset (78.2%) (Fig. 2A). To assess whether there are any significant differences in regional methylation status, the correlation coefficients between the four datasets at both the 100Kb bin and promoter levels were computed. The results revealed a high degree of correlation across all four datasets, with correlation coefficients of at least 0.68 (Fig. 2B,C). The correlation coefficients among the four groups reveal that KM1 and KM2 exhibited the highest correlation, with a coefficient of 0.83 at the 100Kb bin level, and 0.93 at the promoter level. In contrast, the KMB1 and KMB2 had the lowest correlation coefficient, with a value of only 0.68 at the 100Kb bin level and 0.81 at the promoter level (Fig. 2B,C). This suggests that the presence of blood contamination may reduce the stability of the methylation status of kidney marrow.

To further explore the impact of blood contamination on the methylation status of kidney marrow, we compared the distribution of methylation patterns at the regional level for each dataset. From the distribution of regional methylation proportion, the average 5mC methylation proportion of KMB1 and KMB2 were 74.2% and 76.3%, respectively, which were lower than that of the KM group (78.2%) at 100Kb bin level (Fig. 2D). Additionally, the average methylation proportion of KMB1 (approximately 61.7%) is lower than that of KM (approximately 65%) at promoter level (Fig. 2E). Considering the potential influence of read length and read coverage differences among samples on methylation proportion, we calculated the 5mC methylation proportion and read length for each read and subsequently computed the correlation coefficients between these two variables. Our findings revealed that the coefficients were less than 0.2 across all samples, indicating no significant association between read length and 5mC methylation proportion (Fig. S3). Concurrently, we equalized the yield of KM1, KM2, and KMB2 to match that of KMB1 and compared the distribution of regional methylation patterns among the four datasets with equal coverage. Our results demonstrated a pattern similar to that observed when using total reads (Fig. S4). The observation suggests that the KMB samples may have been subject to greater variability in methylation patterns, potentially due to the presence of blood contamination.

### Methylation pattern in eleven different tissues from WGBS

**Fig. 3:**
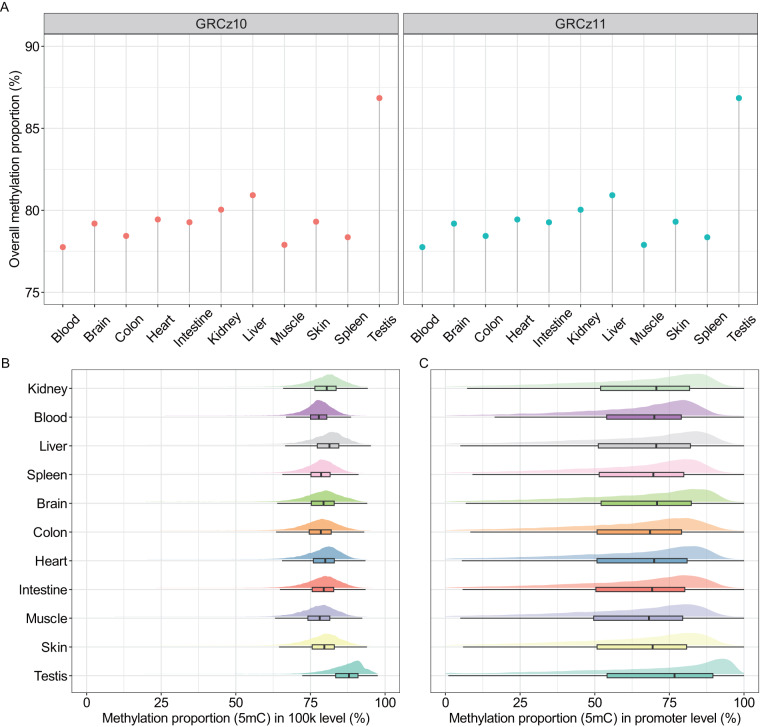
The distribution of methylation proportion across various zebrafish organs using whole-genome bisulfite sequencing (WGBS) datasets. (**A**) The overall methylation proportion of 11 distinct tissues, including kidney, blood, brain, colon, heart, intestine, liver, muscle, skin, spleen, and testis, based on the GRCz10 and GRCz11 zebrafish genome assemblies. (**B**) Distribution of 5mC methylation proportion at 100 kb bin intervals across the 11 tissue datasets based on the GRCz11 zebrafish genome. (**C**) Distribution of 5mC methylation proportion at the promoter level for the 11 tissue datasets, also based on the GRCz11 zebrafish genome.

As is widely recognized, each tissue processes its distinctive methylation pattern. To assess the methylation patterns among different tissues, especially the blood and kidney, we conducted a comparison of the CpG site methylation using the zebrafish tissues datasets from NCBI Gene Expression Omnibus, which included the whole-genome bisulfite sequencing (WGBS) results in eleven zebrafish adult tissues^25^. The liftOver tool (https://genome.ucsc.edu/cgi-bin/hgLiftOver) was utilized to convert the original base-modification profiling data from the GRCz10 genome to the GRCz11 genome, which is necessary to ensure consistency and compatibility of the data with the least version of the genome^14^. Despite the exclusion of a few positions due to differences between the two references, the average overall 5mC methylation proportion was found to be similar to that of the original GRCz10 genome data (Fig. 3A).

As illustrated in Fig. 3B, the average genome-wide 5mC proportion across all tissues ranged from 77.4% to 86.5%. Notably, the liver and testis exhibited a proportion exceeding 80%, indicating a potentially important role of DNA methylation in these tissues. At the promoter level, the average 5mC proportion ranged from 63.2% to 69.6% across all tissues (Fig. 3C). Especially, the 5mC methylation proportion for blood was 77.4% at the genome-wide level and 64.7% at the promoter level. In contrast, the kidney exhibited a 5mC methylation proportion of 79.6% at the genome-wide level and 65% at the promoter level. This discrepancy may explain why KMB has a lower 5mC level than that of KM.

### Comparison of WGBS and ONT methods in methylation calling

**Fig. 4:**
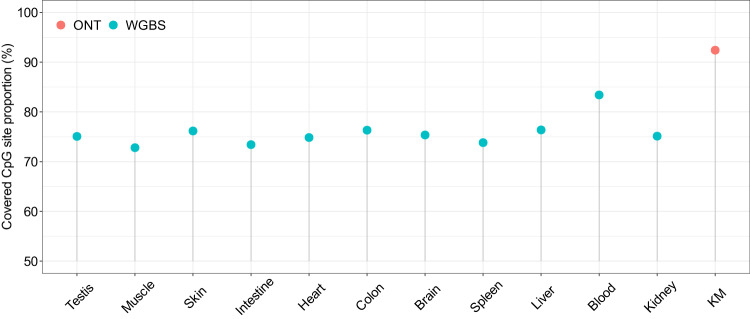
Comparison of CpG site recover proportion between WGBS and ONT datasets. The ONT dataset is represented by red points, while the WGBS datasets are represented by green points. The full CpG site number (100%) for the 25 chromosomes in the zebrafish genome is 23,591,891.

Next, we compared the efficacy of two distinct methods, WGBS and ONT, in calling methylation in CpG sites. Taking into account the fact that the ONT method can identify the single 5mC methylation proportion with directionality, we counted both the C-base (in the forward direction) and the adjacent G-base (which is essentially equivalent to the C-base in the reverse direction) as a single CpG site (see method for detail). As shown in Fig. 4, the ONT method was able to cover 92.4% of CpG sites with at least one positive and revered read pair across the 25 zebrafish chromosomes (GRCz11), while the WGBS method covered a less proportion of CpG sites, ranging from 70% to 80%, with a maximum of 83.4%. This observation suggests that the datasets generated using the ONT platform exhibit a higher rate of genome recovery compared to those generated using WGBS.

**Fig. 5:**
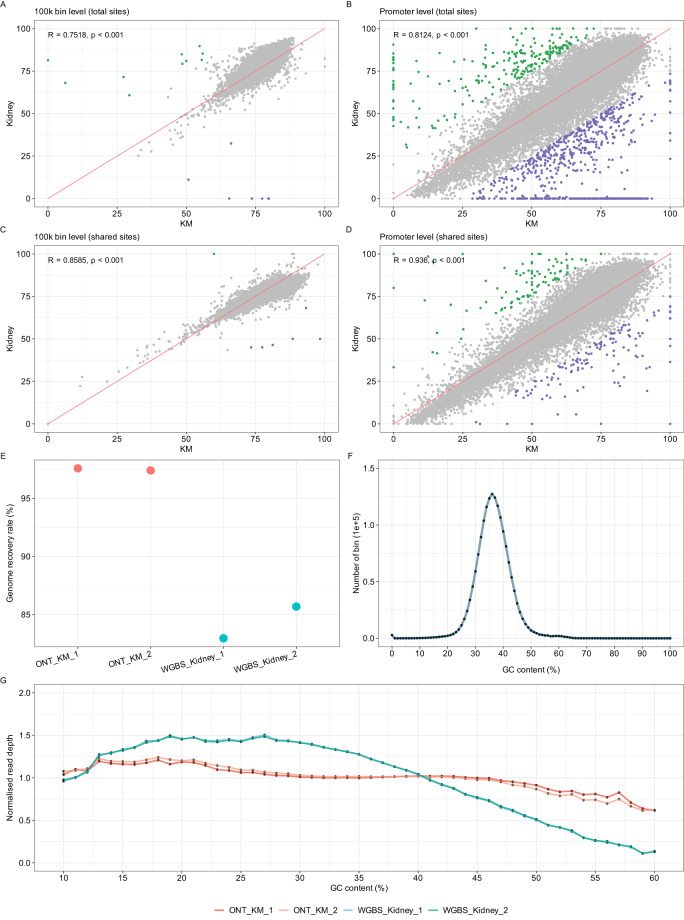
Comparison of GC bias between ONT and whole-genome bisulfite sequencing (WGBS) platforms. (**A**) Pearson correlation analysis for all genomic sites with 100 kb intervals between KM and kidney tissue. (**B**) Pearson correlation analysis of the gene promoters between KM and kidney tissue. (**C**) Pearson correlation analysis for shared sites with 100 kb intervals between KM and kidney tissue using the shared sites from ONT and WGBS. (**D**) Pearson correlation analysis of the gene promoters between KM and kidney tissue using only the shared sites from ONT and WGBS. The red line represents the diagonal in (**A**–**D**). (**E**) The genome recovery rate for the four datasets. (**F**) GC content distribution of zebrafish genome using 1 kb bins. (**G**) The read coverage bias in ONT and WGBS in relation to GC content with a bin size of 1 kb for this plot. A normalized coverage closing to 1 indicates no or low coverage bias. KM, kidney marrow. ONT, Oxford Nanopore Technologies. WGBS, whole-genome bisulfite sequencing.

To further validate the reliability of our ONT sequencing data, we compared the genome-wide and promoter CpG methylation levels of kidney marrow with those profiled from kidney tissue. We initially included all the CpG sites reported in each dataset and grouped them into 100Kb bins to estimate the correlation coefficient (R) at both the genome-wide and promoter levels. The resulting R values were 0.75 and 0.81, respectively (Fig. 5A,B). As the WGBS datasets covered fewer CpG sites than ONT datasets, we also selected the shared positions in both the WGBS and ONT data to evaluate the correlation at the whole-genome and promoter levels. As shown in Fig. 5C,D, this approach resulted in an improvement in the correlation coefficient in both the 100Kb bin and promoter levels. To further explore the relationship across different tissues and sequencing platforms, we computed the correlation coefficient of regional methylation using the shared sites between Blood (WGBS), Kidney (WGBS), KMB1 (ONT), and KMB2 (ONT). Our results indicated the two samples from WGBS exhibited the highest R values, while the two KMB samples had the lowest. Additionally, although the R values between Kidney and KMBs were high, they were still lower than that between KM and Kidney (Fig. S5). These observations suggest that the presence of blood in kidney marrow may affect the methylation pattern.

To explore the reduced CpG recovery rate in the WGBS dataset and the discrepancies between ONT and WGBS, we assessed potential sequencing bias in genomic regions with varying GC content.

To address this question, we compared four datasets, including kidney sample 1 (WGBS)^26^, kidney sample2 (WGBS)^26^, KM1(ONT), and KM2 (ONT), which had a raw yield of approximately 47.4 Gb, 70.9 Gb, 20.6 Gb, and 20.5 Gb, respectively. It is noteworthy that both WGBS datasets exhibited a lower genome recovery rate of approximately 85%, which is lower than that of ONT datasets (approximately 98%), despite higher data yield than ONT data (Fig. 5E). This indicates the presence of bias in the WGBS method.

To determine whether the observed bias in the WGBS method is due to the GC content, further investigation is needed. As illustrated in Fig. 5F, the majority of GC content in the zebrafish genome lies between 10% and 60%, which guided our focus for read coverage analysis within this range. Our result demonstrated that ONT reads were relatively uniformly distributed across the genome, irrespective of GC content (Fig. 5G). Conversely, WGBS reads displayed a distinct bias, characterized by increased read depth in regions with low GC content and decreased read depth in regions with high GC content (Fig. 5G). This GC bias may lead to uneven genomic coverage when characterizing DNA methylation using WGBS reads, particularly in high GC content regions.

### Supplementary information

supplementary table and figures

## Data Availability

All software utilized in this study is publicly available, and their respective parameters are detailed in the Methods section. In cases, where no parameters were specified, default values, as recommended by the software developers, were employed. The scripts utilized in this study are accessible at https://github.com/lrslab/Zebrafish-Multisequencing.
