# Supplementary material for: Unraveling the whole genome DNA methylation profile of zebrafish kidney marrow by Oxford Nanopore sequencing
Source: Sci Data. 2023 Aug 10;10:532. doi: 10.1038/s41597-023-02431-5 (PMC10415270; doi:10.1038/s41597-023-02431-5)
Supplement: Supplementary file 1 — supplementary table and figures [file 41597_2023_2431_MOESM1_ESM.docx]

**Supplementary Table.1 Statistics of raw and clean data from four datasets.**

| Sample | Total read number | Total base number | N50 (bp) | GC content (%) | Type |
| --- | --- | --- | --- | --- | --- |
| KMB1 | 1,480,526 | 7,274,665,362 | 9,701 | 37.41 | raw data |
|  | 1,337,171 | 6,520,871,435 | 9,847 | 37.24 | clean data |
| KMB2 | 1,147,916 | 9,890,958,127 | 16,158 | 37.3 | raw data |
|  | 997,838 | 8,750,122,360 | 16,425 | 37.06 | clean data |
| KM1 | 3,376,221 | 20,565,279,276 | 12,345 | 36.91 | raw data |
|  | 3,035,164 | 18,952,883,564 | 12,706 | 36.77 | clean data |
| KM2 | 1,932,515 | 20,532,184,192 | 20,665 | 36.59 | raw data |
|  | 1,831,917 | 19,735,312,599 | 20,894 | 36.55 | clean data |

KMB1: kidney marrow with blood 1; KMB2: kidney marrow with blood 2; KM1: kidney marrow 1; KM 2: kidney marrow 2


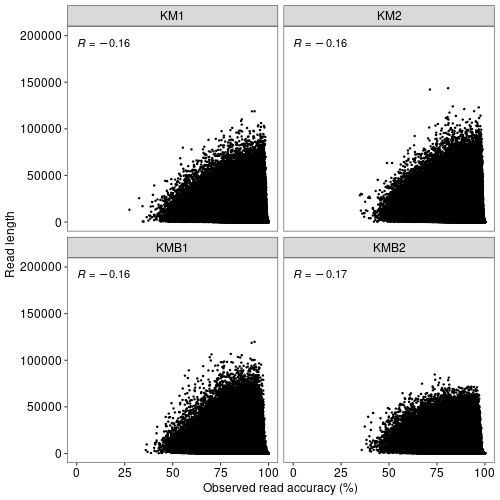


**Figure S1.** Correlation between read length and observed read accuracy in four datasets. Each point represents a single read.


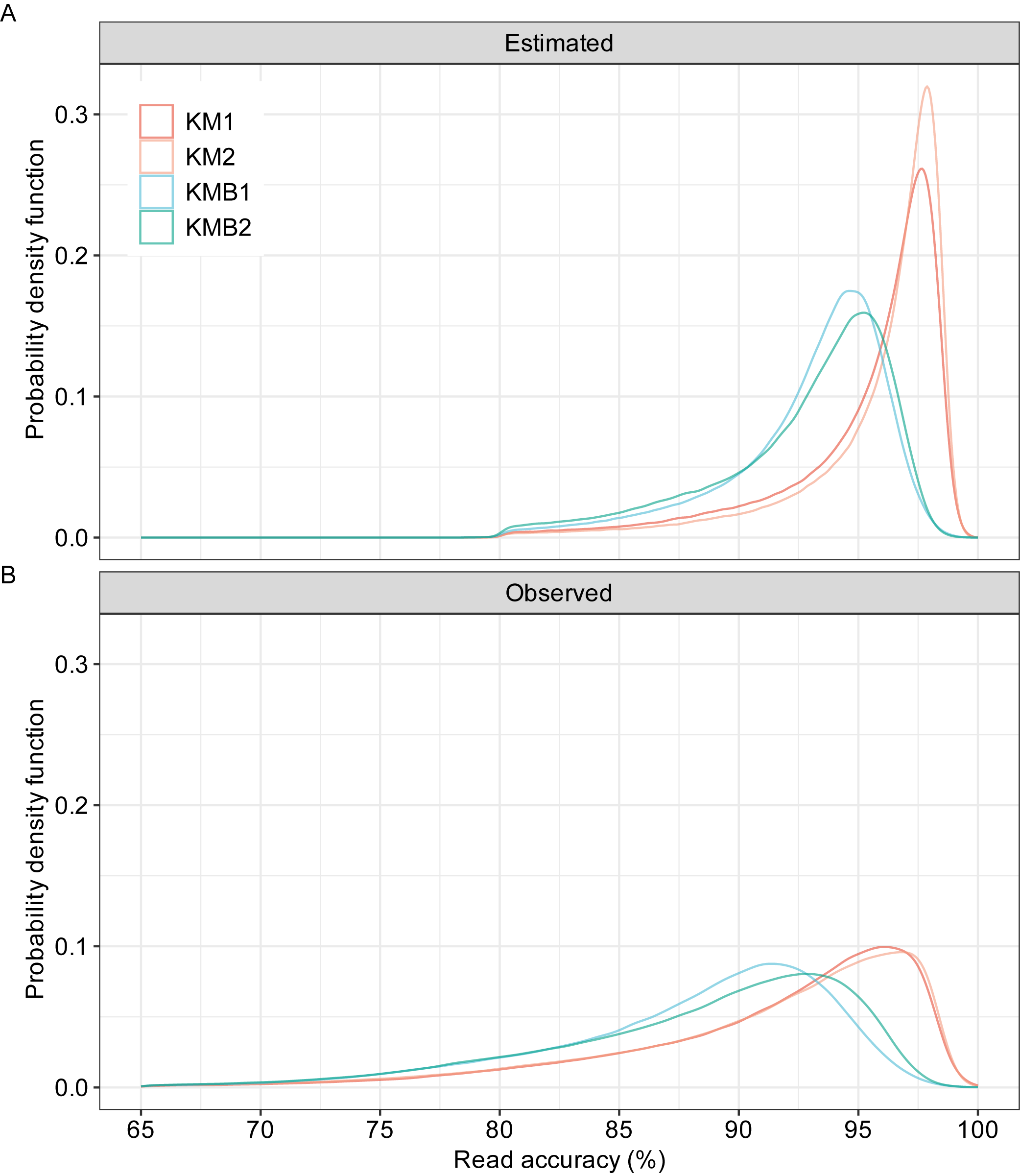


**Figure S2. Comparison of four sample datasets with the same read coverage: KM1, KM2, KMB1, and KMB2.** The read coverage of KM1, KM2, and KMB2 was subsampled to match that of KMB1. (A) Density distribution of estimated read accuracy. (B) Density distribution of observed read accuracy for mapped reads. KM, kidney marrow. KMB, kidney marrow with blood.


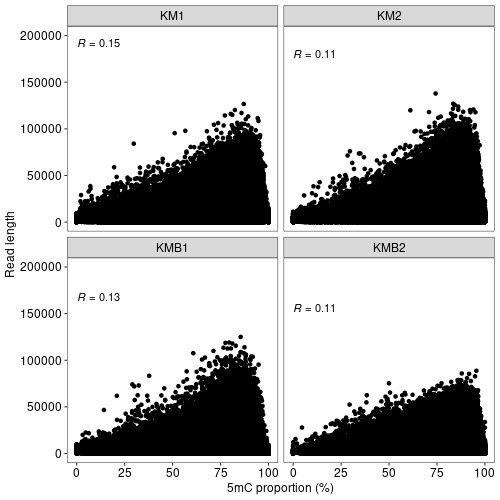


**Figure S3.** Correlation between read length and 5mC proportion at CpG sites in four datasets. Each point represents a single read.


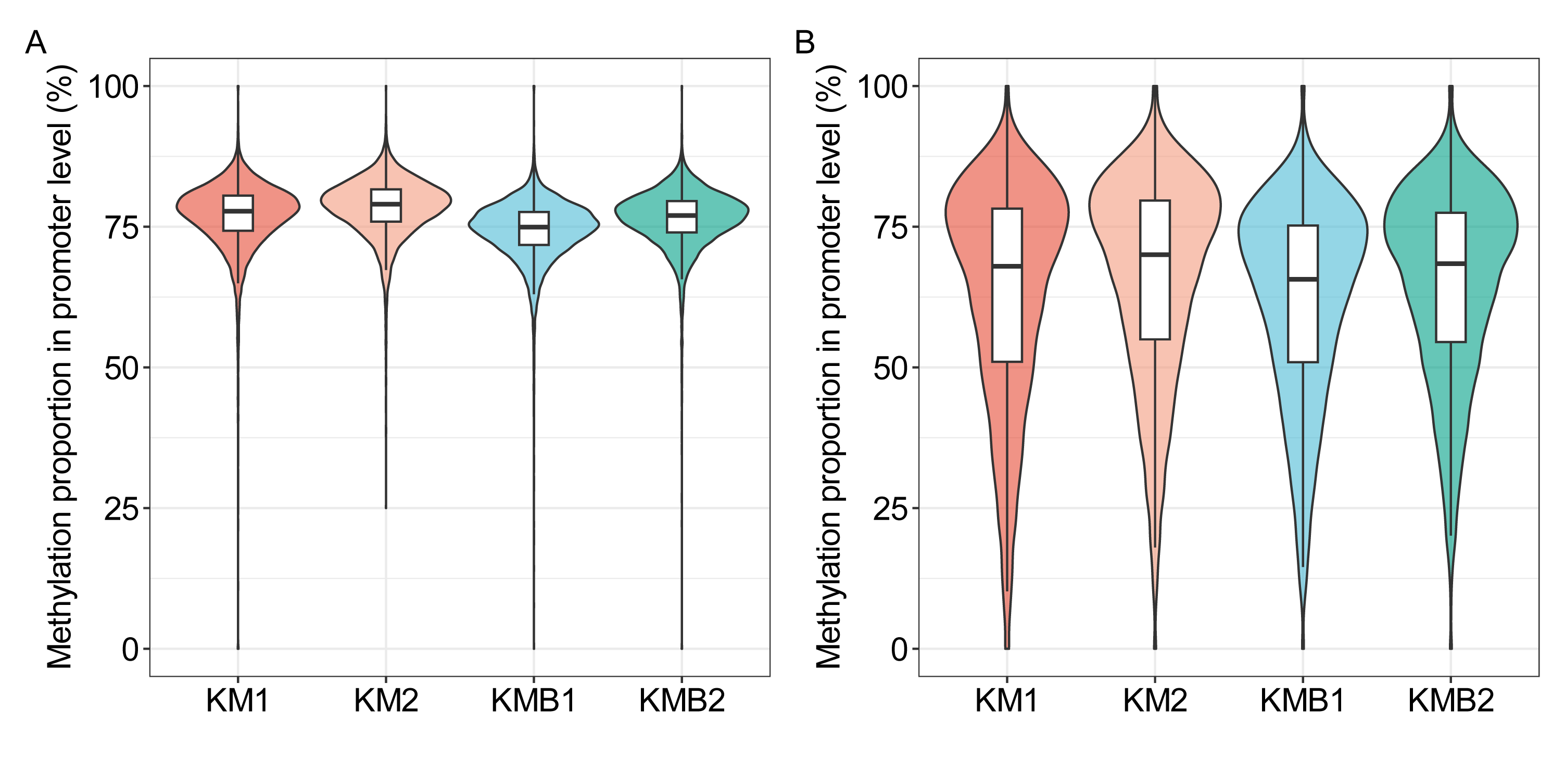


**Figure S4. Comparison of four sample datasets with the same read coverage: KM1, KM2, KMB1, and KMB2.** The read coverage of KM1, KM2, and KMB2 was subsampled to match that of KMB1. (A) Methylation distribution of the four datasets at the 100 kb bin intervals. (B) Methylation distribution of the four datasets at the promoter level.


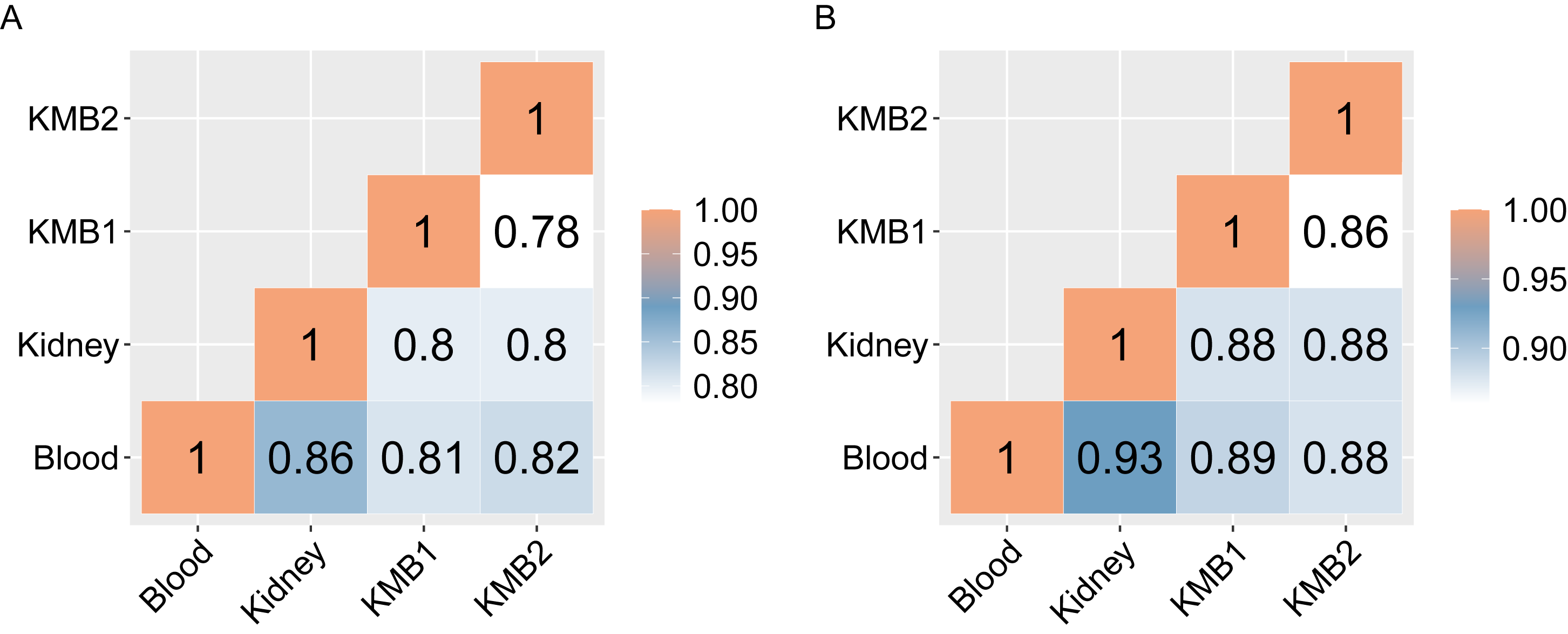


**Figure S5. Correlation of methylation proportion in four datasets including KMB1 (ONT), KMB2 (ONT), Blood (WGBS), and Kidney (WGBS) using the shared sites.** (A) Pearson correlation coefficients between the four datasets in the 100 kb bin intervals. (B) Pearson correlation coefficients between the four datasets in the promoter regions. ONT, Oxford Nanopore Technologies. WGBS, whole-genome bisulfite sequencing.
